# Highly pathogenic avian influenza A(H5N8) outbreaks: protection and management of exposed people in Europe, 2014/15 and 2016

**DOI:** 10.2807/1560-7917.ES.2016.21.49.30419

**Published:** 2016-12-08

**Authors:** Cornelia Adlhoch, Ian H. Brown, Svetla G. Angelova, Ádám Bálint, Ruth Bouwstra, Silke Buda, Maria R. Castrucci, Gavin Dabrera, Ádám Dán, Christian Grund, Timm Harder, Wim van der Hoek, Katalin Krisztalovics, Frances Parry-Ford, Rodica Popescu, Anders Wallensten, Anna Zdravkova, Siamak Zohari, Svetla Tsolova, Pasi Penttinen

**Affiliations:** 1 European Centre for Disease Prevention and Control (ECDC), Stockholm, Sweden; 2 Animal and Plant Health Agency (APHA), Weybridge, United Kingdom; 3 National Centre of Infectious and Parasitic Diseases, Sofia, Bulgaria; 4 National Food Chain Safety Office (NEBIH), Budapest, Hungary; 5 GD Animal Health Deventer, Netherlands; 6 Robert Koch Institute (RKI), Berlin, Germany; 7 Istituto Superiore di Sanita (ISS), Rome, Italy; 8 Public Health England (PHE), London, United Kingdom; 9 Friedrich-Loeffler-Institut (FLI), Federal Research Institute for Animal Health, Greifswald-Insel Riems, Germany; 10 National Institute for Public Health and the Environment (RIVM), Bilthoven, the Netherlands; 11 National Center for Epidemiology, Budapest, Hungary; 12 National Institute of Public Health, Bucharest, Romania; 13 The Public Health Agency of Sweden, Stockholm, Sweden; 14 Bulgarian Food Safety Agency, Sofia, Bulgaria; 15 National Veterinary Institute (SVA), Uppsala, Sweden

**Keywords:** Avian influenza, A(H5N8), 2016, outbreaks, EU/EEA, infection control

## Abstract

Introduction of highly pathogenic avian influenza (HPAI) virus A(H5N8) into Europe prompted animal and human health experts to implement protective measures to prevent transmission to humans. We describe the situation in 2016 and list public health measures and recommendations in place. We summarise critical interfaces identified during the A(H5N1) and A(H5N8) outbreaks in 2014/15. Rapid exchange of information between the animal and human health sectors is critical for a timely, effective and efficient response.

## Avian influenza A(H5N8) situation in Europe, December 2016

In September 2016, the Food and Agriculture Organization (FAO) of the United Nations raised awareness for the potential reintroduction of highly pathogenic avian influenza (HPAI) virus A(H5N8) to Europe after the detection in a wild swan in the Tyva Republic, Russia, in June 2016. A potential spread of the virus was assumed via the migratory bird routes of duck, geese and swans [1]. The communication followed earlier reports in 2016, of A(H5N8) in wild and domestic birds in the Republic of Korea, and Taiwan, and the event suggested re-introduction of the virus via wild birds migrating back to Europe for overwintering.

### Outbreaks in wild birds

From 30 October to 6 December 2016, 14 European countries (Austria, Croatia, Denmark, Finland, France, Germany, Hungary, the Netherlands, Poland, Romania, Russia, Serbia, Switzerland, and Sweden) as well as Egypt, India, Iran, and Israel reported HPAI A(H5N8) outbreaks in domestic poultry or detections in wild or zoo birds (Figure) [2]. Tunisia and Ukraine reported HPAI A(H5) outbreaks suspected to be A(H5N8). Since the first finding in October, the virus spread rapidly across central Europe. It mostly affected wild water birds, but also birds of prey that feed on dead birds’ carcasses. Infections of the latter indicate a recent introduction into the local resident bird population.

**Figure:**
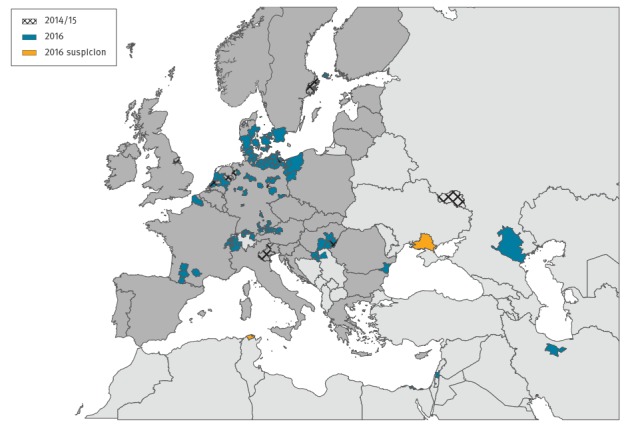
Detection of highly pathogenic avian influenza virus A(H5N8) in wild birds and poultry, Europe and neighbouring regions, November 2014 to February 2015 and October to December 2016^a^

EU/EEA: European Union/European Economic Area.

^a^ The map displays the situation as at 6 December 2016. Dark grey represents the EU/EEA.

### Outbreaks in poultry holdings

In 2016, outbreaks in poultry holdings were reported from Austria, Denmark, France, Germany, Hungary, the Netherlands, Poland and Sweden [2]. This resembles the situation in the northern hemisphere winter 2014/15 when a virus of the same clade 2.3.4.4 caused outbreaks in six European countries (Germany [3,4], Italy [5], Hungary [6], the Netherlands [7], Sweden and the United Kingdom [8]), mainly in closed poultry holdings, and sporadic detections in wild birds and a zoo [2,3]. Although the viruses belong to the same genotype clade, viruses during the 2014/15 outbreak belonged to a different group of clade 2.3.4.4, group A (Buan-like), while the current 2016 viruses cluster in clade 2.3.4.4 group B (Gochang-like).

This report presents critical points identified during the HPAI A(H5N1) and A(H5N8) outbreaks in 2014/15 for preparedness, communication and public as well as animal health recommendations and measures to contain outbreak of avian influenza.

## Potential risks to human health

No human cases of influenza A(H5N8) virus infection have been reported despite large numbers of people being occupationally exposed while managing the avian outbreaks, thus the risk for humans is considered very low [9]. This contrasts with the risk of bird-to-human transmission of influenza A(H5N1) and is likely due to A(H5N8) receptor-binding properties with the latter virus being better adapted to avian-like receptors than human-like receptors [8,10-12]. Although the sequence information available for the haemagglutinin and neuraminidase proteins of recent A(H5N8) isolates does not show any evolution towards increased affinity for humans, these viruses should be closely monitored for any adaptation [13]. The non-structural protein (NS) gene of the A(H5N8) virus detected in a wild sea duck, common Goldeneye, in Sweden in mid-November is truncated (217aa) and reassortment in polymerase acidic (PA) and nucleoprotein (NP) genes has been observed compared to those viruses detected earlier in June in Tyva (S. Zohari, personal communication, December 2016; sequences available in GISAID: EPI863862-69; National Veterinary Institute; Uppsala, Sweden A/Common Goldeneye/Sweden/SVA161117KU0322/SZ0002165/2016).

Influenza viruses undergo constant reassortment. Recent human cases of influenza A(H5N6) reported from China illustrate how A(H5) viruses belonging to the same clade 2.3.4.4 as A(H5N8) viruses, can gain the ability to infect humans without any of the major adaptation processes referred to above. The current properties of the virus are not suggestive of pandemic risk. Still, the likely lack of immunity in humans against A(H5N8) and its increasing geographic distribution and incidence in animals justify constant monitoring of outbreaks in birds. Current concerns among veterinarians include the potential ability of A(H5N8) to infect mammals such as cats and dogs: thus precautions should be put in place to minimise the risk of exposure for these animals.

## Available guidance on protective measures

Although the risk of human infection is considered very low [14], most of the available national guidance documents recommend a number of risk mitigation measures to minimise exposure. They target different groups: (i) for the general population recommendations are to avoid exposure to potentially infected birds by not touching dead wild birds, and instead inform local veterinary authorities; (ii) local public and veterinary health authorities are recommended to limit the number of individuals exposed to birds suspected or confirmed to have HPAI; and (iii) individuals exposed occupationally are recommended to use appropriate personal protective equipment (PPE).

## Experiences from 2014/15 outbreaks and measures in 2016

In October 2015, animal and public health experts involved in the HPAI A(H5N1) and A(H5N8) outbreaks in Europe in 2014/15, reviewed relevant national protocols available in European Union/European Economic Area (EU/EEA) countries, actions implemented and lessons learnt, in a workshop organised by the European Centre for Disease Prevention and Control (ECDC) [15].

The Table summarises key recommendations from selected public health authorities that managed avian influenza outbreaks in recent years and are valid in 2016. Generally they contain strict use of PPE when handling potentially infected birds, carcasses or other material, with some flexibility based on the local risk assessment in some countries. Post-exposure prophylaxis with neuraminidase inhibitors is advised, often based on individual clinical assessment or local risk assessment. Some, but not all countries recommend pre-exposure prophylaxis that is to be continued during and after exposure. All countries recommend follow-up, passive or active, of those exposed, for development of symptoms for the duration of the maximum incubation period estimated to be around 7-10 days. Exposed people with influenza-like symptoms according to the EU case definition [16] should immediately be tested for influenza virus infection, preferably using lower respiratory tract specimens and including H5-specific tests. Healthcare workers managing suspect human cases should take appropriate contact and airborne precaution measures (Table).

**Table:** Avian influenza prevention and control measures implemented by selected national European Union public health authorities^a^, December 2016

Measure	Bulgaria	England	Germany ^a^	Hungary	Italy ^b^	Netherlands ^c^	Romania	Sweden
**Protection of exposed individuals during avian influenza incidents**	Individuals exposed occupationally or in close contact with infected or potentially infected birds should use suitable PPE such as disposable overalls, nitrile/vinyl gloves, rubber boots, goggles, and a filtering half mask with exhalation valve. The personnel handling infected or potentially infected birds should observe the biosecurity instructions for collection and disposal of the equipment used.	Individuals exposed occupationally should use appropriate PPE: disposable or polycotton overall, disposable gloves, rubber or polyurethane boots, FFP3 respirator with exhalation valve and close fitting goggles.	PPE including disposable gloves, clothing, headwear, protective boots, close fitting goggles and masks: FFP1 if aerosolisation is not likely, otherwise FFP3 with exhalation valve	Full body protection: (overall, gloves, boots, goggles) and FFP3 respirator	Occupationally exposed individuals should use appropriate PPE: FFP3 masks, rubber gloves and boots resistant to detergents/disinfectants, disposable overall and hair cover, eye protection.	Eye protection, FFP2 mask (for cullers FFP3), boots, disposable overall, hair cover, disposable gloves	Measures for protecting the individuals who come in contact with infected birds or likely to be infected, birds alive or dead are: PPE and appropriate conditions for collection, neutralisation and storage of the equipment used.	Individuals occupationally exposed should use appropriate PPE: disposable or polycotton overall, disposable gloves, rubber or polyurethane boots, FFP3 respirator with exhalation valve and close fitting goggles.
**Period that exposed people should be monitored for symptoms**	7-10 days	10 days	7 days	10 days	Up to 10 days following exposure	Poultry workers / cullers are requested to report symptoms until 10 days post exposure, to the municipal health service, i.e. passive monitoring, which can be scaled up to active monitoring.	7 days	10 days
**Testing**	Clinical specimens (naso-pharyngeal swabs/ bronchoalveolar lavage fluid/endotracheal aspirate/ pleural fluid/ sputum) from exposed people with respiratory symptoms in close contact with ill or dead birds, their family members or travellers to countries with registered avian influenza cases will be collected to identify influenza and specifically A(H5).	Influenza and specifically A(H5) from respiratory tract samples	Influenza and specifically A(H5) from respiratory tract samples; if serological testing is considered paired, serum samples should be collected.	Test for human influenza A,B,C and influenza A/H5; respiratory tract and paired (10-14 days) serum samples should be taken and sent to reference laboratory of NCE.	Influenza and A/H5 from respiratory tract samples; paired serum samples should also be taken.	Testing only after telephone consultation with the virologist on duty (24/7) at RIVM; nose, throat, and eye swab for PCR analysis.	Naso-pharyngeal swabs will be collected to identify avian influenza virus A(H5).	Influenza and specifically A(H5) from respiratory tract samples
**Pre or post-exposure chemoprophylaxis**	Exposed individuals: antiviral chemoprophylaxis, immediately after exposure	Individuals who are exposed occupationally may be offered antiviral chemoprophylaxis as an added precaution following an appropriate risk assessment and according to defined algorithm.	Exposed individuals with direct contact to infected birds following an appropriate risk assessment (for example appropriate PPE during exposure or not)	Post-exposure prophylaxis: oseltamivir antiviral prophylaxis for 10 days for occupationally exposed individuals	Exposed individuals on evaluation by local health authorities	All exposed workers, farmers, and their family members; a national supply of antivirals is kept at RIVM.	Specific measures to protect exposed individuals: prophylactic antiviral treatment for 7 days, immediately after exposure	Individuals who have been exposed without wearing protective equipment depending on the type of avian influenza and the exposure
**PPE and other precaution measures to be used by healthcare workers assessing symptomatic, exposed people**	PPE: disposable gloves; single use mouth/nose mask, goggles; standard, contact and airborne precautions	Contact and airborne precautions; this includes eye protection, FFP3 respirator, gowns and gloves when working in same room as the symptomatic person.	Standard, contact and airborne precautions; eye protection	Standard, contact and droplet-airborne precautions with eye protection	Standard, contact and airborne precautions, including eye protection	Standard PPE and personal hygiene measures	PPE: single use gowns, single use mouth/nose mask, goggles, single use gloves; standard contact and airborne precautions	Standard, contact and airborne precautions; eye protection; gowns and gloves when working in same room as the symptomatic person
**Seasonal influenza vaccine recommendation**	Risk population groups recommended by WHO for influenza vaccination	As per usual annual recommendations for at-risk groups	As per recommendation of the German standing committee for vaccination (STIKO), including poultry workers	Based on the recommendation of NCE (in the annual circular of the Chief Medical Officer): poultry/pig workers (breeders, transporters, cullers, workers in processing plants, etc.)	Poultry/pig workers and healthcare workers	Only vaccinated workers can be involved in culling. Vaccination is offered to other workers, farmers and their family members if outbreaks occur during influenza season.	Risk population groups recommended by WHO for influenza vaccination (including HCW) and for exposed individuals, in order to avoid the reassortment between human and avian virus	As per usual recommendations, currently no requirement for poultry workers
**Measures (applied or planned) to follow-up on exposed individuals during current A(H5N8) outbreaks or detections in birds**	NA	Exposed individuals will be followed up either actively or passively during incident and for 10 days after last exposure. Choice of active or passive follow-up depends on type of exposure and an assessment of use of protective measures, including chemoprophylaxis if indicated.	UNKN	Identification of individuals exposed and / or involved in the culling; individuals with specific conditions (underlying diseases, immunodeficiencies, pregnancy, individuals older than 62 years of age) should be excluded from culling. Information of exposed individuals about the risk, the signs and symptoms of the disease and the methods of prevention. Assigning a physician responsible for monitoring the health of the exposed for 10 days.	NA	UNKN	UNKN	Passive surveillance of those exposed without proper protective equipment
**Seroepidemiological follow-up planned in 2016/17**	No	Not routinely done; focus is on investigation of symptomatic individual patients exposed during incidents.	No	No	No	In case of an outbreak there is a research protocol which includes serology at T0 and T4weeks – with analysis for different human, avian, swine influenza viruses using microarray.	No	No
**Other studies planned related to current outbreaks**	No	Will be determined on an incident-by-incident basis, if required to support the public health response	UNKN	In order to assess the efficacy of disinfection, 100 g dust samples are taken. Wetted tissue swabs are applied on 900 cm^2^ surface from different parts of the pen representing the whole area.	No	Possible air sampling in and around poultry farms in case of a new outbreak	No	No

FFP: filtering face piece; NA: not applicable; NCE: National Centre for Epidemiology; RIVM: National Institute for Public Health and the Environment; PPE: personal protective equipment; RIVM: Rijksinstituut voor Volksgezondheid en Milieu (The Dutch National Institute for Public Health and the Environment); UNKN: unknown; WHO: World Health Organization.

^a^ The information provided applies generally, but each incident is assessed individually to ensure a fully appropriate response.

^b^ National guidelines in Italy leave flexibility to provide antiviral prophylaxis during avian influenza outbreaks, whereas vaccination with seasonal vaccine is annually recommended for veterinary service and poultry/swine industry workers.

^c^ National guidelines in the Netherlands leave considerable flexibility regarding monitoring, antiviral prophylaxis and seasonal vaccination. The approach is tailor-made to ensure a rapid and flexible response to any signal.

The experts concluded that the actions taken during the 2014/15 outbreaks were adequate to prevent human cases, but some challenges and discrepancies were noted. There was agreement that timely sharing of information between the animal and human health sectors as well as between countries is crucial for an appropriate and early response. Intersectoral communication should also continue between outbreaks to foster cooperation at national level. Most countries appear to use a maximum level of precaution during incidents rather than basing precautions on a careful risk assessment, and experts concur whether this was the most efficient approach. Although a general overview of published evidence was considered useful, risk should be assessed locally.

Recommendations on use of antivirals or seasonal vaccines differed between countries. Some challenges were encountered when post-exposure prophylaxis was recommended, but sufficient antivirals were not immediately available. The experts suggested that rapid availability of antivirals in each country should be reviewed and ensured.

Recommendations for seasonal influenza vaccination of poultry workers in general differ between countries. Seasonal influenza vaccination of exposed individuals during an outbreak was suggested in most countries to avoid co-infection with seasonal and avian influenza viruses which could be followed by reassortment events. However, England considered vaccination with seasonal influenza vaccine during an avian influenza outbreak as being too late for exposed individuals to develop an antibody response necessary for individual protection.

Active follow-up of exposed individuals is resource-demanding and requires a risk assessment. Suggestions from the meeting were to develop a tool to track and trace information on detections and outbreaks in animals as well as related human exposure and follow-up measures, in real time.

Streamlined messages based on evidence and targeted to those concerned are necessary. Communication barriers i.e. language were identified as reason for failure to follow up exposed mobile and migrant workers on poultry farms. This could be remedied by providing leaflets in different languages.

Large farms might have better safety and training standards than small farms, but response capacity and timeliness during outbreaks may still be insufficient.

Rapid communication and sharing of the viral genetic information is important to estimate the reliability of the PCR-based A(H5) HA gene detection applied in each country/region and antiviral treatment efficacy.

## Conclusions

Humans have been and will be exposed to influenza A(H5N8) virus from infected birds, their carcasses or contaminated material in the coming weeks in Europe. Although no human cases of influenza A(H5N8) have been documented, expert advice is that precautionary measures should be taken to minimise human exposure and possible infections. Relevant guidance and protection measures have proven sufficient during the avian influenza outbreaks in 2014/15 but were critically reviewed and adjusted where necessary. Well-designed follow-up studies among the exposed would help to document the (lack of) risk from A(H5N8) to humans and the effectiveness of control measures.

Timely communication between the animal and human health sectors is vital to enable a rapid, effective and efficient response to the ongoing outbreaks. Any human infection with a novel influenza subtype should trigger an immediate international notification through the International Health Regulations (IHR) mechanism and the EU Early Warning and Response System.
